# Principles guiding the restoration of the root-filled tooth

**DOI:** 10.1038/s41415-025-8401-4

**Published:** 2025-04-11

**Authors:** Shanil R. Patel, Callum Youngson, Fadi Jarad

**Affiliations:** 550640202279863964730 Specialist in Endodontics, Endo 61 Dental Practice, 61 Church Road, Gately, Cheshire, SK8 4NG, United Kingdom; 942924274410759037142 Emeritus Professor, Liverpool Dental School, Faculty of Health and Life Sciences, University of Liverpool, L3 5PS, United Kingdom; 231158481397096629631 Professor and Honorary Consultant in Restorative Dentistry, Department of Restorative Dentistry, University of Liverpool School of Dentistry, Liverpool, United Kingdom

## Abstract

Endodontic treatment is usually required because of significant coronal disease or traumatic tissue loss. The restoration of the subsequently endodontically treated tooth is also complicated by the reduction in its structural strength consequent to accessing the pulp chamber and the removal of radicular dentine during root canal instrumentation, alongside some alteration of dentinal properties during disinfection by chemical agents, prior to obturation. A loss of proprioceptive feedback, which may lead to increased loading, can place further stress on the already very compromised structure. This article considers the principles of restoring endodontically treated teeth: assessing restorability, providing a coronal seal to prevent reinfection, and gaining retention for a core where necessary, to restore aesthetics and function. Consideration is given to the patient and material factors that influence the decision to restore the treated tooth using direct or indirect restorations. Specific attention is given to anterior or posterior teeth and the various materials which may be used in their overall restoration with their associated, probable, longevity.

## Key points

- After determining restorability and subsequently endodontically treating teeth, the clinician should determine the likely forces that will be exerted on the tooth in function and, potentially, parafunction.
- A restorative material should be selected based on these forces, the remaining tooth structure, and patient acceptability, to provide an antimicrobial seal for the root canal filling and provide protection to the underlying tooth structure.
- A direct or indirect restoration constructed from an appropriately selected material, based on patient and material factors, should restore aesthetics and function with a reasonably predictable longevity.

## Introduction

The European Society of Endodontology has published a consensus document summarising the views of an expert committee to provide clinical recommendations regarding the restoration of the root-filled tooth, which is intended to be updated in light of future developments.^1^ However, as early as 1974, Ramfjord proposed that ‘the purpose of restorative dentistry was to restore and maintain health and functional comfort of the natural dentition combined with satisfactory aesthetic appearance'.^2^ When restoring endodontically treated teeth, Bhuva *et al*.^3^ have noted that the restoration should additionally prevent microbial leakage into the root canal system and protect the residual tooth structure against further tissue loss.

While it is accepted that endodontically treated teeth are more vulnerable to fracture than intact teeth, the most significant factor affecting their strength would appear to be the volume of remaining natural tooth substance.^4^^,^^5^^,^^6^^,^^7^ Endodontic treatment also usually requires removal of some tooth structure that may reduce the strength of the remaining tooth. While this can be limited to radicular tissue (in the case of a traumatically exposed pulp), in most cases, access cavities are prepared, often in previously restored teeth. Coronal, as well as radicular, tissue is removed to gain adequate access for instrumentation, disinfection and obturation, and this further compromises the structural integrity of the tooth.^8^

It has long been recognised that small changes in dentinal moisture content can also occur following endodontic treatment^9^ but these do not, of themselves, result in dentine, which is significantly more brittle.^10^ However, some diminution in dentine properties has been observed associated with materials often used in root canal treatment eg sodium hypochlorite (NaOCl) and calcium hydroxide (CaOH).^11^

As well as the intra-operative changes noted above, the loss of pulp vitality may result in greater loads being applied to the tooth than would be found in a vital tooth (due to the loss of proprioceptive feedback originating from the pulp)^12^ and this may further predispose the root-filled tooth to fracture. It should be noted that the type and timing of the final restoration (which should follow the principles of Ramfjord and Bhuva *et al*.)^2^^,^^3^ also contribute significantly to the tooth's survival.^13^^,^^14^

While the principles of restoration of both anterior and posterior endodontically treated teeth are the same, the emphasis can vary, as anterior teeth experience different loading, play a variable role in mandibular guidance, and usually have a higher aesthetic requirement than posterior teeth. However, before considering the tooth position within the arches, it is worth first considering whether the decision to carry out root canal treatment (in the best interests of the patient) is justified.

## Restorability of the tooth

An assessment should be made of the likely success of the proposed treatment. As well as endodontic considerations, many patient factors, including wishes, expectations, financial status, oral health status, and response to stabilising treatments, can help to indicate the patient's ability and motivation to maintain the restored tooth. More specific factors, such as the strategic importance of the tooth (contribution to aesthetics and/or function, its periodontal status, and the need to maintain the tooth as an abutment for a current or future prosthesis) should also be considered. When these are all favourable, a more detailed analysis of the affected tooth is important, as well as planning for the final restoration. A systematic review^15^ has considered the seven tooth-related factors found to affect the prognosis of endodontically treated teeth (Table 1).

**Table 1:** Key prognostic factors for ETPT identified from a systematic review^15^

**Factor**	**Comment**
Remaining coronal tooth structure	Significant correlation with prognosis
Ferrule	Inconclusive evidence*
Crown/root ratio	Inconclusive evidence*
Tooth type	Premolars associated with greater survival than molars
Location	a) Mandibular and maxillary ETPT have similar prognoses. b) Terminal tooth (in either arch) has poorer survival
Periodontal status	Some evidence that greater periodontal disease associated with poorer prognosis
Number of proximal contacts	a) Premolars: significant correlation with prognosis b) Molars: less conclusive except for terminal tooth (see ‘location')
Cracks	Low correlation, but poorer prognosis when extending into pulp floor or root canal
Key: * = In the absence of conclusive evidence, it is advised that these factors are included in considerations for restoring the tooth until more definitive evidence becomes available	

In terms of restorability, an early index was proposed to help the clinician determine the likely ability of the tooth to either retain a coronal restoration, or else adopt an alternative approach, such as a post-retained core, or crown lengthening.^16^ More recently, a Dental Practicality Index considers the many factors noted above, as well as the tooth-specific elements of the earlier index to provide a more holistic guide to clinical treatment decision-making.^17^ This latter index has also been reviewed favourably, retrospectively, in other studies to determine its applicability and validity in retreatment cases.^6^^,^^18^ When the decision is made to root-fill and restore the tooth, the principles underpinning the success of the treatment should be considered in turn.

## Prevention of microbial leakage into the root canal system

As well as the loss of the dental pulp leading to the loss of dentinal reparative processes, the subsequent absence of dentinal tubule fluid outflow in a non-vital tooth allows microorganisms to enter the dentinal tubules and potentially reinfect the root canal system.^19^ The final coronal restoration plays a crucial role in preventing reinfection of the disinfected and obturated root canal system, and thus increasing the prognosis, by providing a hermetic seal to prevent microbial reinfection from the oral cavity.^20^ This ‘coronal sealing' of exposed (coronal or radicular) dentinal tubules can be gained by using adhesive techniques, extending restorations over the dentine when using a ferrule (see later), or a combination of these techniques.

To create a complete seal, the tooth would ideally have multiple layers of endodontic/restorative protection, each acting as an independent barrier to recontamination:

- The root canal filling should be well-condensed to the ideal working length, with a non-shrinking sealer (such as a hydraulic calcium silicate sealer) to avoid wash-out or voids that may allow microbial re-entry
- Consideration should be given to covering the furcation and the coronal extent of the root canal filling with a thin antimicrobial adhesive layer^21^ (which will also resist microbial contamination if a provisional restoration leaks or fails)
- Ideally, an adhesive ‘core' material should be placed eg a high-density glass ionomer or calcium silicate cement, or alternatively, an adhesive and a bulk-fill composite (possibly fibre-reinforced) to completely seal the furcation and entrance to the root canal system and replace lost dentine
- A final restorative layer, provided by a direct or indirect restoration, will replace lost enamel.

If the outer layer of restoration fails, then each successive inner layer acts to protect the root canal filling and facilitate more conservative re-restoration.

## Restoration of form and function

This is key to controlling the occlusal and/or parafunctional loads that the restored tooth will experience. This is particularly important where a post core has been provided, as loading can be transferred mainly to the post, resulting in post fracture^22^ or tooth fracture where there is not a significant ferrule.^23^

**Fig. 1:**
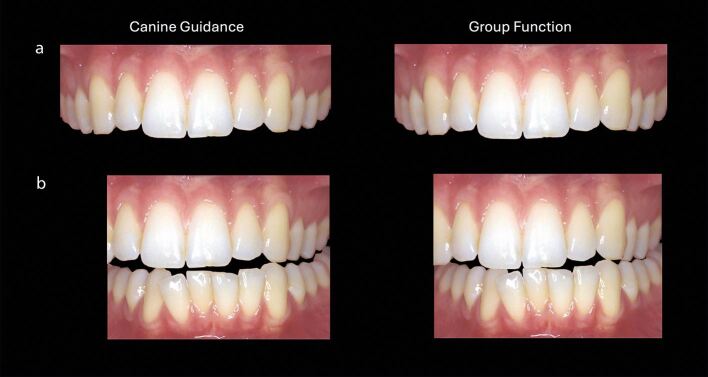
Digital ‘mock-up' illustrating likely consequences of converting canine guidance (the 23) to group function on a) aesthetics and b) functional contacts

Where the tooth in question has a potential antagonist, study models or a digital scan of the dentition should be used to determine any occlusal contacts in and between intercuspal position, retruded contact position, and protrusive and lateral excursions, including possible parafunctional movements (evidenced by matching tooth facets) that may load the restored tooth, given that the lack of pulpal mechanoreception can result in even higher forces being exerted than may be expected. In most cases, the endodontically restored tooth should be part of a ‘group function' occlusal scheme rather than taking the guidance alone. This is complicated in the case where the canine is being restored following root canal treatment. To protect the canine from large lateral loads, it is often advisable (unless only a small access cavity has been required in an otherwise unrestored tooth) to convert this from a canine-guided to a group function scheme. However, this can have both aesthetic effects, as the canine will tend to be shorter than its contralateral partner, giving a slightly unbalanced smile line against the lower lip, and also possible functional consequences (Fig. 1). With the loss of canine guidance and reduced vertical travel of the mandible in lateral excursion, there is an increased risk of introducing working or non-working side posterior contacts,^24^ which could lead to posterior restoration/cuspal fractures. The best solution is to plan the proposed changes using physical (or digital) models to analyse the aesthetic and functional effects and assess the planned changes in provisional restorations, refining the occlusal scheme if necessary, to achieve the desired result, before reproducing this successful form in the definitive restoration.

## Restoring satisfactory aesthetics

This is harder to define as the perception of aesthetics can vary from individual to individual (clinician and, most significantly, the patient).

Many simple access cavities can be restored satisfactorily by composite resin restorations, but when the restoration extends onto labial/buccal aspects, shade-matching becomes critical. As well as the restoration shape, shade and surface, the ‘framing' of the tooth by the gingival architecture can strongly affect the aesthetic result, so there should be well-documented discussions with the patient regarding the likely outcome, and their understanding of required compromises, before commencing treatment.

## Protection of the residual tooth structure

As noted previously, the greatest risk to the successfully endodontically treated tooth arises from forces that will fracture the tooth where its structural integrity has been compromised to a greater, or lesser, extent by endodontic access and preparation.^25^ While it may be considered logical that more conservative forms of access will result in more durable endodontically treated teeth, especially where marginal ridges can be retained intact, there is no strong evidence currently available to support this hypothesis and, in fact, the effects upon the ability to satisfactorily clean, disinfect and obturate the root canal system may have greater consequences for survival of the tooth.^26^^,^^27^

The number of remaining walls of the tooth can help determine whether a simple intracoronal restoration is likely to be sufficient.^28^^,^^29^ Although this has not been widely investigated in anterior teeth, the suggestion has been made that if only minimal restoration of the palatal, and one proximal, surface is required, a simple composite will tend to be sufficient. However, increasing the number of involved surfaces to include both proximals, especially if these restorations are large, tends to indicate that a full crown should at least be considered, with even more compromised surfaces strongly indicating that form of treatment.

To protect posterior teeth from cuspal flexure and resultant tooth fracture, especially where an extensive mesial-occlusal-distal cavity will exist after endodontic access, some form of cuspal protection is indicated. This will be considered later when discussing posterior teeth considerations.

## Maintaining health

As with all other tooth tissue, there is an ongoing risk of dental caries and so all post-endodontic restoration margins should fit accurately, prevent overeruption of opposing teeth and be designed to minimise plaque accumulation, with appropriate tooth contacts and emergence profiles to harmonise with the surrounding natural dentition. This will also help to minimise the consequences upon the gingival health, or periodontal health, where a restoration has to extend subgingivally to seal the margin.

## Timing of definitive restoration

Although there is limited evidence as to the ideal timing of placing a definitive restoration, there has been the suggestion that the presence of a permanent, rather than a temporary, restoration has a significant effect on endodontic success,^30^ with one retrospective study noting that root-filled posterior teeth receiving a crown, before four months, were three times less likely to be extracted than teeth restored for longer using composite or amalgam restorations.^31^ Given the need to prevent microbial contamination and protect the remaining tooth structure, it is logical, in the absence of any contraindications, to provide the definitive restoration as soon as possible.

## Specific tooth considerations

### Anterior teeth

Anterior teeth are often exposed to non-axial loading and the strength of the tooth cervically, which can resist torquing forces, is very important. Anecdotally, with improved oral health, the number of anterior teeth requiring endodontic treatment due to caries has decreased considerably and so, often, simple palatal access cavities will be able to be restored simply with composite resin restorations, which both adequately seal and restore the tooth to similar strengths of those found in intact teeth.

However, where large proximal cavities or restorations have also been present, or the access cavity has become large cervically, eg to identify a sclerosed canal orifice, consideration needs to be given to how a final restoration can be retained that restores the tooth to adequate strength to resist functional loading.

Before the widespread adoption of adhesive dental techniques, when it was considered that insufficient coronal structure was remaining, a conventional approach was to remove coronal tissue to allow a cast post and core to be constructed. Traditionally, the coronal structure was often reduced to a shape that would allow a stone die to be reliably constructed, before post canal preparation and recording of an impression. While early cast post and core designs often resulted in a completely decoronated tooth, it was subsequently recommended that coronal tissue be retained to allow a ‘ferrule effect',^23^ where the overlying crown allowed functional forces to be transferred partly to the tooth substance rather than being borne entirely by the post/tooth interface. A minimum of 1 mm ferrule for cast post and cores was advocated initially, with subsequent investigators noting that up to 3 mm of retained coronal buccal dentine improved in vitro fracture resistance.^32^ Given the role of anterior teeth in occlusal guidance, the clinician should take account of the likely loading applied to the retained tooth surface (eg buccal and lingual/palatal) when deciding how much tissue to retain for a ferrule.

## Post-retained restorations

Early concepts suggested that a post may reinforce root-treated teeth, but it is now clear that removing tooth tissue to retain a post weakens the remaining structure,^8^ and even adhesively luted posts do not reinforce the root.^33^ While metallic post systems are now used less frequently, they offer some ‘retrievability' should they fracture,^22^ whereas ceramic or zirconium posts are virtually impossible to remove if fractured, and so use of these latter materials as posts should be avoided.^33^

The principal aims of any post is to retain as much tooth structure as possible, provide a microbial seal and retain a core. While they are very seldom required in the restoration of molars, posts are more often used in anterior and premolar teeth, in cases where there is inadequate coronal structure to retain the core.^34^ In these cases, the post system selected should match the post-treatment root canal morphology as closely, and disrupt the obturation, as little as possible, while mirroring the biological shape of the root structure. For this reason, threaded or parallel posts, which provide the greatest post retention but cause stress accumulation within the root^35^ (and are more likely to result in lateral perforation during post channel preparation),^33^ should be avoided.

**Fig. 2:**
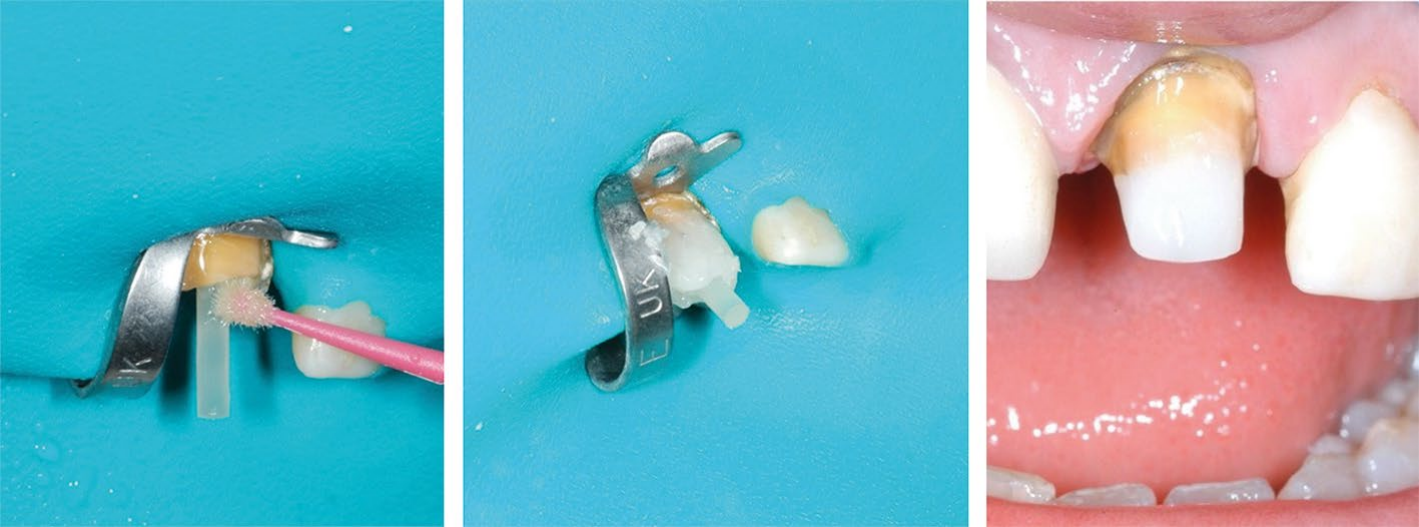
Fibre-post retained composite core on the 21, with ferrule for final crown

The principle of retaining as much coronal structure as possible has evolved with the widespread adoption of the use of adhesive composite materials to reinforce that which remains; although, adhesively luted fibre posts do not reinforce tooth structure. However, these do help to retain a composite core and provide benefits where coronal tooth structure provides a ferrule^36^ (Fig. 2).

Many older, laboratory-based, in vitro studies focused on the beneficial mechanical (retention and stress distribution) characteristics of longer posts. In gaining these mechanical advantages, there was often less consideration given to the detrimental effects upon the microbial seal of removing much of the root canal filling, or the effects on the root structure,^8^ to place a traditionally cemented post. However, current adhesive techniques, which maintain more coronal tissue, allow clinicians to gain an adequate length of post with less disturbance of the root canal filling, as well as maintaining an antimicrobial seal. Fibre posts are also less likely to cause root fractures compared with metal posts, due to their modulus of elasticity being similar to that of dentine - an obvious advantage. While traditional systems suggested that 1 mm thickness of remaining dentine thickness was required in axial walls,^37^ modern systems can maximise retention of coronal structure, thereby providing a longer ferrule and more favourable stress distribution.

## Anterior crowns

**Fig. 3:**
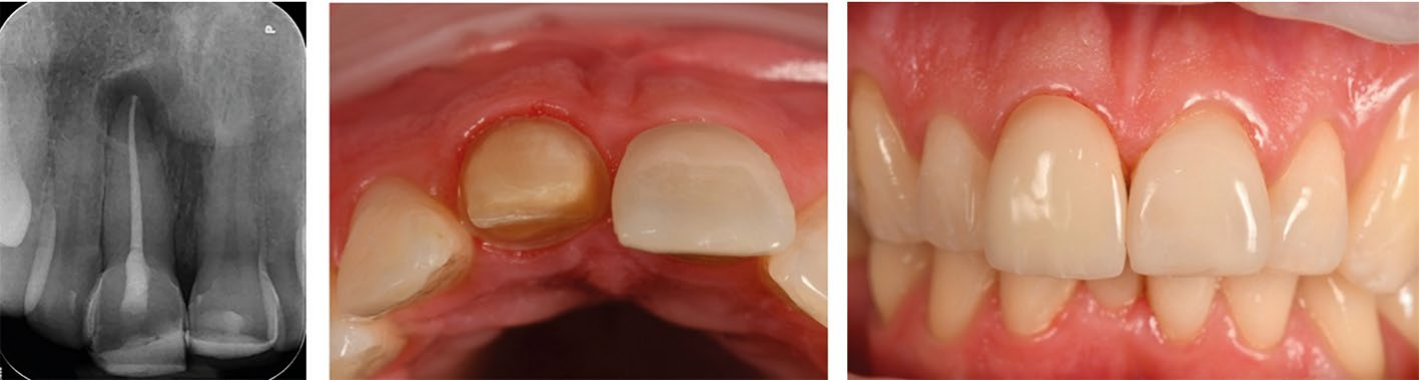
Pressed-glass all-ceramic crown (the 11)

**Fig. 4:**
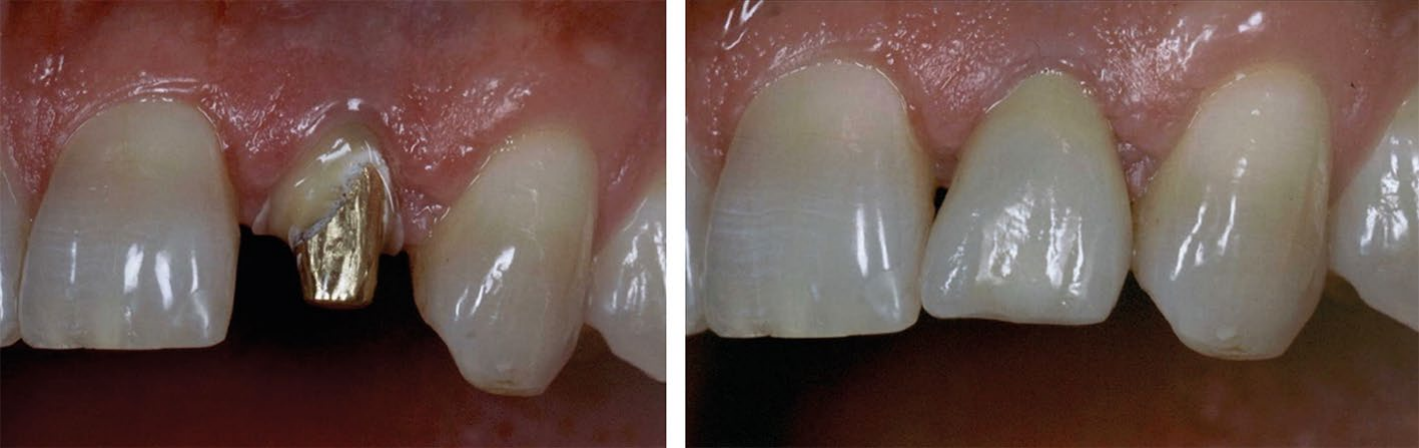
Metal ceramic crown placed over cast-metal post and core (the 22)

This choice of crown material to restore the endodontically treated tooth should, once again, be based on the aim of retaining as much tooth structure as possible and maintaining a microbial seal, while being able to withstand foreseeable loading, restoring form and function, and meeting the aesthetic needs of the patient. Modern pressed-glass or other all-ceramic crowns meet many of these criteria, especially when cemented adhesively to remaining tooth structure (Fig. 3). However, metal-ceramic crowns may still be indicated where more durability is required, due to high functional/parafunctional loading, or where a cast metal post and core system is present, which would limit the achievable aesthetics and adhesive bonding of an all-ceramic crown (Fig. 4).

Regardless of the crown material, the preparation should aim to maximise the ferrule effect while not compromising the biological width any more than is necessary, to distribute forces favourably to the root surface and allow a sympathetic emergence profile to be developed.

### Posterior teeth

The same principles of maintaining a microbial seal while restoring form, function and aesthetics, apply to restoration of anterior and posterior teeth but, in the latter case, there are differences that make their timely restoration more critical. The magnitude of applied forces, exerted mainly axially, is much greater. The functional cusp-fossa relationship can exert lateral loading on non-functional cusps, which have often been weakened by previous restorations and/or endodontic access. If marginal ridges are also lost, tooth stiffness can be reduced by up to 63%.^38^ Molar teeth tend to be most susceptible to caries and cracks^39^ and are generally considered more complex teeth to treat and restore.^40^ Multiple studies have shown that root canal treatment accounts for a low proportion of treatment failure.^38^^,^^41^^,^^42^^,^^43^ Tooth-related factors that affect the prognosis of endodontically treated posterior teeth (ETPT) have been considered in a systematic review and meta-analysis^15^ and these are summarised in Table 1. However, the correct choice of coronal restoration can reduce the risk of failure caused by one or more of these factors.

## Direct restorations

Although they require significant skill from the providing clinician, direct restorations are usually less invasive, do not require a laboratory stage and are cheaper for the patient. However, when comparing survival rates of direct and indirect restorations, it is important to differentiate between restoration survival and tooth survival. One large (n = 13.9 million) study to assess restoration longevity (in both vital and non-vital anterior and posterior teeth, treated under the prevailing NHS contract)^44^ found that, although crowns had a better restoration survival rate at 15 years compared with composite restorations, tooth survival was very similar. A systematic review also concluded that there was insufficient evidence to conclude that crowns had a greater effect on tooth survival that conventional fillings.^45^

One (single operator, practice-based, retrospective) study has noted that the annual failure rate of composite (0.86%) restorations was lower than that of crowns (2.63%) after 8.7 years.^46^ Another has reported no significant difference between fibre-post and composite or fibre-post, composite and crown restorations over a three-year period.^47^

Composites are easily mouldable, aesthetically excellent, have a similar modulus of elasticity to dentine than metals and ceramics, and can be repaired easily. Short-fibre reinforced composite has been shown to have improved load-bearing capacity and crack-arresting mechanisms in laboratory-based studies.^48^^,^^49^^,^^50^ However, further evidence is required regarding tooth survival when comparing indirect restorations and direct restorations which incorporate fibre-reinforced composites.

Although many endodontically treated teeth will have previously lost one or more marginal ridges, one group has concluded that, although crowns improved fracture resistance, composite restorations placed on endodontically treated teeth with both marginal ridges retained, and a maximum of two surface losses, had comparable survival rates after five years (composite 88.5%; crowns 95%),^51^ supporting the use of conservative, direct restorations where the marginal ridges are intact.

## Indirect restorations

When restoring the posterior tooth with an indirect restoration, a number of factors^52^ come into play, including a complex interaction of considerations for the clinician (Box 1).

There is a consensus that indirect restorations increase the survival of root-filled teeth. One retrospective study noted that the five- and ten-year survival with a crown was 94% and 89%, respectively, but without a crown, these figures dropped to 77% and 62%.^13^ In other previously mentioned studies, posterior teeth without a crown were approximately six times more likely to require extraction after eight years^14^ and the presence of a crown following root canal treatment had a significant effect on tooth survival.^5^

Box 1 Clinical considerations when restoring an ETPT

- Functional and aesthetic requirements, which affect the choice of material
- Thickness of remaining tooth walls (and those which will remain after preparation for the definitive restoration)
- Resistance form of the final preparation
- Finish line (eg butt-end, rounded shoulder, chamfer, knife edge)
- Margin length (and position)
- Number of barriers that will be in place to preserve the microbial seal
- Likelihood of achieving good moisture control, especially for adhesive luting cements
- Likelihood of increasing wear to any antagonist tooth

## Onlay restorations

Onlays can be a valid alternative to crowns as they allow for a more conservative tooth preparation, while still providing cuspal coverage to protect the underlying tooth structure; although, they do have a longer restoration margin length than a crown to seal (and keep under clinical review). They are often considered more difficult to prepare as there is the need to avoid undercuts, while conserving as much tooth structure as possible, and the marginal finishing lines are less ‘templated' than those of a crown preparation. Depending on the choice of material, there may also be a greater or lesser aesthetic compromise as the onlay tooth interface is usually visible but, posteriorly, this may be less of a concern for patients, and a sizeable proportion of dentists would still choose a gold restoration in selected cases.^52^ With respect to the tooth-coloured onlay materials, one systematic review concluded that there was inconclusive evidence regarding which restoration survived longer when comparing resin and ceramic onlays or inlays.^53^

Survival studies for onlays focused solely on ETPT are sparse. In a retrospective study of gold restorations (including onlays) on mainly posterior teeth, authors found very high survival rates at 30 years, regardless of restoration design.^54^ Although only 10% were endodontically treated, they noted that ETPT was a risk factor to survival. A 2016 systematic review and meta-analysis of the survival rate of resin and ceramic inlays, onlays and overlays found that ceramic onlays had ten-year survival rates of 91%.^55^ However, this study was also not limited to ETPT. Further evidence is required when comparing onlays and crowns as restorations of choice for ETPT, but currently, it seems that onlays have survival rates comparable to crowns while being more conservative of tooth structure.

## Endocrowns

**Fig. 5:**
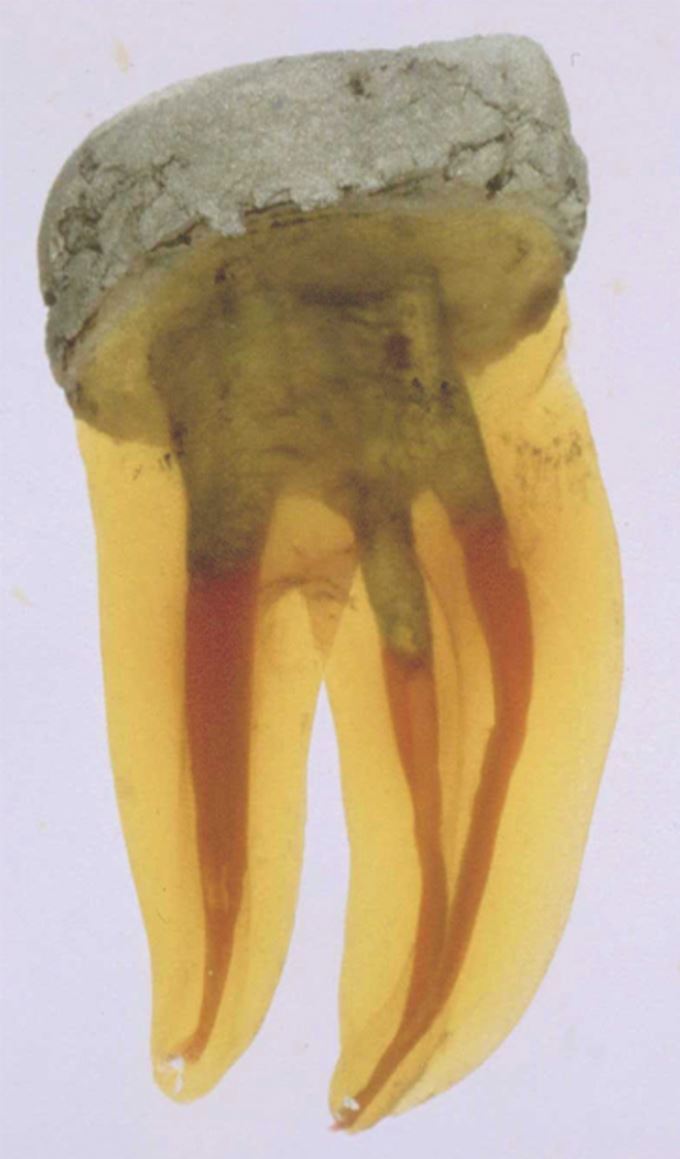
Amalgam Nayyar core in cleared molar. Image reproduced with permission from M. Howdle *et al.*, ‘Microleakage of adhesive and conventional Nayyar cores', *Quintessence International*, 2002

Endocrowns are lab-made, full-coverage crowns with an integrated intracoronal extension^7^ and so could be considered an extreme form of onlay. They incorporate macromechanical retention through anchorage within the pulp chamber and micromechanical retention from adhesive cementation.^56^ While benefiting from retaining as much coronal tissue as possible and avoiding the need for post-channel preparation, they offer fewer barriers to microbial seal compared to a ‘Nayyar'^57^ form of retained core. This latter technique was originally designed to gain macromechanical retention for an amalgam core using the pulp chamber and coronal 2-4 mm of the root canals of posterior teeth (Fig. 5). More recent adaptations of this technique use tooth-coloured core materials with adhesive techniques to also enhance the microbial seal. The final crown, with a cervical finishing margin, will then provide both resistance form and a further marginal seal.

## Crown materials

The major factors in the selection of a material of choice for restoration of ETPT are summarised in Table 2. It should be borne in mind that a thorough clinical assessment is essential when selecting the most appropriate crown material for each individual case.

**Table 2:** Material and technical factors influencing the selection of an indirect restoration

**Material**	**Advantages**	**Disadvantages**	**Survival rates**	**Considerations**	**Preparation techniques**
Metal-ceramic	Acceptable aesthetics High strength and toughness Good support for veneering porcelain Low cost Well-established technique	Not usually as aesthetic as all-ceramic options (lack translucency) Metal can show through cervically, or if the porcelain chips Exposed opaceous porcelain can wear opposing teeth	79% - 20 yrs^62^	Most common posterior crown material due to strength, aesthetics and cost Suitable for most patients, including those with bruxism May use polished metal surfaces on high-wear areas	Traditional preparation Requires adequate reduction for both metal and porcelain layers in areas of aesthetic need
Zirconia monolithic or layered/veneered	Good aesthetics (esp. layered/veneered options) Strongest and most fracture-resistant ceramic (flexural strength ~1,200 MPa) Low plaque accumulation Biocompatible and tissue-friendly (polished, unglazed)	Layered/veneered zirconia prone to chipping and micro-cracks (esp. with reduced core support or in bridges) Can be more difficult to adjust and polish	97.3% - 5 yrs (zirconia-based)^68^ 97.4% - 5 yrs (metal-based with over-pressed veneers)^68^	Monolithic zirconia preferred for ETPT due to reduced risk of chipping More studies needed with larger sample sizes and longer follow-up periods to provide definitive longevity	Traditional preparation (may be able to use more minimal techniques eg vertiprep^60^ or ‘biologically oriented preparation technique'^61^ - but only very limited evidence available for the efficacy of these)
Glass ceramics: lithium disilicate (eg IPS E.max) leucite-reinforced (eg IPS Empress)	Excellent aesthetics - translucency mimics natural teeth Can be used both anteriorly and posteriorly Conservative preparations possible	Lower fracture resistance than zirconia, esp. posteriorly May not be suitable for patients with bruxism or parafunctional habits	97.1% - 11 yrs (IPS Empress II and IPS E.max Press)^70^	Suitable for ETPT in specific cases with high aesthetic need Survival rates may vary between materials Careful case selection is important	Traditional preparation Adequate reduction needed to accommodate chosen material with adequate strength
Gold	Long survival times Excellent wear resistance Conservative preparations - strong in thin sections Biocompatible and tolerated by gingival tissues Does not wear opposing teeth Easily adjusted and polished	Compromised aesthetics Increasingly a loss of technician expertise High cost	Very high survival rates at 30 years^55^ 70% - 20 yrs (gold crowns)^74^	Excellent option for non-aesthetic zones, esp. in parafunction May be a good choice for patients with limited interocclusal space	Traditional preparation Specific finishing margin requirements for optimal gold adaptation

### Metal ceramic

The high strength and toughness of a metal core, and the support provided to the veneering porcelain coupled with low cost and acceptable aesthetics, warrants the selection of metal-ceramic crowns (MCCs) as the most commonly chosen posterior crown material.^58^^,^^59^ One, practice-based study of anterior and posterior teeth assessed the survival rates of ETPT restored with MCCs as 79% at 20 years.^62^

### Zirconia

**Fig. 6:**
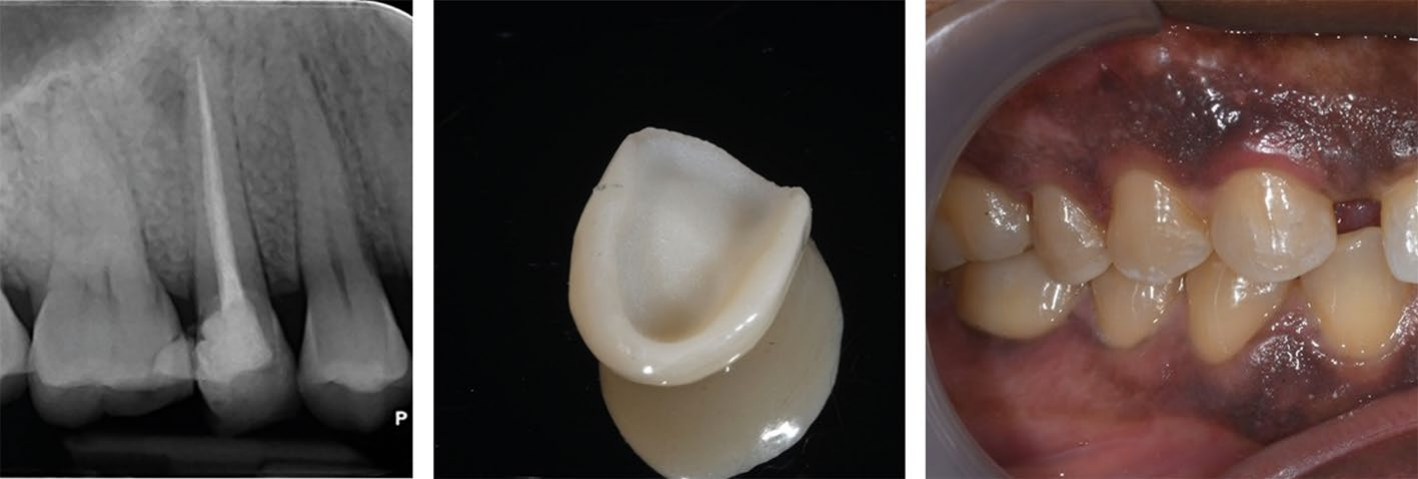
Monolithic zirconia onlay on the 15 providing cuspal protection (buccal margin visible below cusp tip)

Zirconia is the strongest and most fracture-resistant ceramic available. In its tetragonal form, it has a flexure strength around 1,200 MPa and fracture toughness greater than 5 MPa.^63^ It also exhibits the lowest plaque accumulation of any ceramic.^64^ Zirconia crowns can come in different forms - monolithic or layered/veneered.^65^ The addition of a separate veneering ceramic to improve aesthetics can lead to future failure of the crown through chipping and micro-cracks, more commonly in bridgework,^66^ or where the underlying zirconia core offers less support to the overlying veneer.^67^ Currently published studies examining performance of this material have excluded patients who parafunction, so this would need to be considered when prescribing this material. Monolithic zirconia is less prone to micro-fracture and chipping (which can occur with the veneered ceramic) and so may be a more suitable option for ETPT and offer an alternative to metal or metal-ceramic crowns (Fig. 6).

A five-year *in vivo* randomised controlled trial, assessing the difference in outcome between zirconia-based and metal-based crowns with over-pressed veneers on ETPT, found five-year survival rates of 97.3% and 97.44%, respectively,^68^ but further studies with larger sample sizes and longer follow-up periods are required to better assess the adequacy of zirconia crowns for restoring endodontically treated molar teeth.

### Glass ceramics

These materials have excellent aesthetic characteristics and so may be selected in patients where there is a high aesthetic need (Fig. 3); although, there is conflicting evidence about their strength and fracture resistance in the posterior segment, especially when compared with zirconia counterparts.^69^ However, one retrospective survival study, with over half involving posterior or endodontically treated teeth, assessed both IPS Empress II and IPS E.max Press (Ivoclar Vivadent, Liechtenstein) crowns over an 11-year period, provided by the same clinician in private practice.^70^ The cumulative survival probability for posterior teeth was 97.1%. Patients with parafunctional habits (23.5% of the sample) were included in this study, and only one crown failed in a bruxist.

There are a variety of glass-ceramic materials available. Survival rates may differ between the different materials,^71^^,^^72^^,^^73^ and evidence tends to be retrospective. Nonetheless, it appears that glass-ceramic restorations have a slightly lower survival rate compared to other crown materials but are still a suitable material for EPTP in specific cases.

### Gold

Gold crowns have been around for decades and are commonly associated with long survival times, excellent wear resistance and conservative preparations. However, the provision of these has reduced due to the compromised aesthetic result, as well as the demanding technique required for optimal tooth preparation and gradual loss of technician expertise, as laboratories move to more ceramics-based infrastructures.

**Fig. 7:**
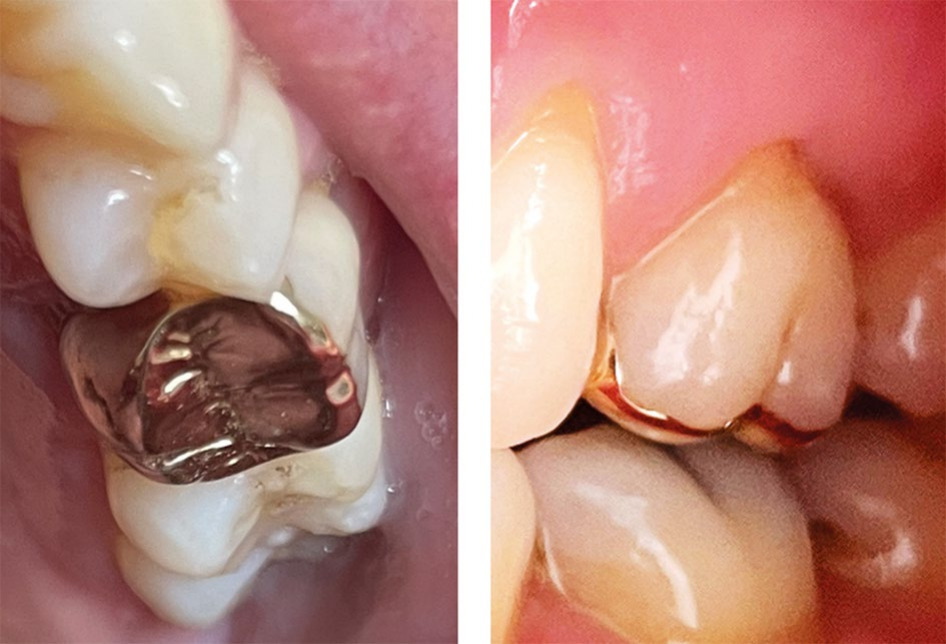
Endodontically treated 26 restored with 3/4 veneer gold crown (retaining as much buccal tooth structure as possible)

Within the literature, there is a lack of evidence specifically focusing the survival of ETPT restored with gold crowns. However, as noted previously, tooth failure due to endodontic reasons is relatively uncommon, so it is worth considering restoration survival rates of gold crowns on vital teeth, with one retrospective study of cast gold restorations finding very high survival rates at 30 years^55^ and another finding gold crown survival rates of 70% at 20 years for posterior teeth.^74^ One *ex vivo* study involving endodontically treated premolars even found that gold onlays were better at resisting cusp fracture than natural tooth tissue!^75^ Although gold restorations may not be aesthetically optimal, they are still considered an excellent option in a non-aesthetic zone (Fig. 7), especially in parafunctional patients.^76^

## Conclusion

Decision-making in the restoration of endodontically treated posterior teeth has moved more from predominantly mechanical considerations towards a more biologically based process. The wide variety of restorative materials and adhesive techniques now available enable clinicians to produce predictably long-lasting, highly aesthetic and functional results, which maintain as much natural tooth structure as possible while providing a good antimicrobial seal to the endodontically treated tooth.
